# MinION-based long-read sequencing and assembly extends the *Caenorhabditis elegans* reference genome

**DOI:** 10.1101/gr.221184.117

**Published:** 2018-02

**Authors:** John R. Tyson, Nigel J. O'Neil, Miten Jain, Hugh E. Olsen, Philip Hieter, Terrance P. Snutch

**Affiliations:** 1 Michael Smith Laboratories and Djavad Mowafaghian Centre for Brain Health, University of British Columbia, Vancouver, British Columbia, Canada V6T 1Z4;; 2 Michael Smith Laboratories, University of British Columbia, Vancouver, British Columbia, Canada V6T 1Z4;; 3 UC Santa Cruz Genomics Institute and Department of Biomolecular Engineering, University of California, Santa Cruz, California 95064, USA;; 4 Department of Medical Genetics, University of British Columbia, Vancouver, British Columbia, Canada V6T 1Z3

## Abstract

Advances in long-read single molecule sequencing have opened new possibilities for ‘benchtop’ whole-genome sequencing. The Oxford Nanopore Technologies MinION is a portable device that uses nanopore technology that can directly sequence DNA molecules. MinION single molecule long sequence reads are well suited for de novo assembly of complex genomes as they facilitate the construction of highly contiguous physical genome maps obviating the need for labor-intensive physical genome mapping. Long sequence reads can also be used to delineate complex chromosomal rearrangements, such as those that occur in tumor cells, that can confound analysis using short reads. Here, we assessed MinION long-read-derived sequences for feasibility concerning: (1) the de novo assembly of a large complex genome, and (2) the elucidation of complex rearrangements. The genomes of two *Caenorhabditis elegans* strains, a wild-type strain and a strain containing two complex rearrangements, were sequenced with MinION. Up to 42-fold coverage was obtained from a single flow cell, and the best pooled data assembly produced a highly contiguous wild-type *C. elegans* genome containing 48 contigs (N50 contig length = 3.99 Mb) covering >99% of the 100,286,401-base reference genome. Further, the MinION-derived genome assembly expanded the *C. elegans* reference genome by >2 Mb due to a more accurate determination of repetitive sequence elements and assembled the complete genomes of two co-extracted bacteria. MinION long-read sequence data also facilitated the elucidation of complex rearrangements in a mutagenized strain. The sequence accuracy of the MinION long-read contigs (∼98%) was improved using Illumina-derived sequence data to polish the final genome assembly to 99.8% nucleotide accuracy when compared to the reference assembly.

Advances in next generation sequencing (NGS) have ushered in a new era of whole-genome analysis. The short sequencing reads generated by sequencing-by-synthesis NGS are well suited for resequencing genomes for which a reference has been established. However, short sequence reads from NGS or Sanger sequencing alone cannot assemble novel complex genomes that contain repeated sequences longer than the reads each of these technologies can span and thus fail to correctly assemble transposable elements and genomic duplications or known genomes with complex chromosomal rearrangements that are often a feature in tumor cells.

To overcome the challenges of assembling complex genomes, the reference sequences for most large genomes were constructed using a hierarchical clone-based strategy. Clones containing genomic DNA were restriction digest-‘fingerprinted’ to assemble a physical map of overlapping clones that were sequenced to generate a contiguous reference genome. For example, the assembly of the *C. elegans* reference genome was made possible by the construction of a physical map of cosmids and yeast artificial chromosomes (Coulson et al. 1988, 1991, 1995) that were individually shotgun-sequenced and manually finished to bridge gaps and ambiguous regions to generate a complete reference genome (The *C. elegans* Sequencing Consortium 1998). This strategy is both labor-intensive and error-prone, as repetitive repeats can be deleted within the clones, together with an inability of short sequencing reads to span large repeat regions. Long-read sequencing platforms present an alternative strategy for the de novo assembly of complex novel genomes.

Long-read single molecule sequencing technologies have increased read lengths 100- to 1000-fold compared to NGS platforms and can span much larger repeat regions than NGS. Until recently, the major platform of this type was the Pacific Biosciences Single Molecule Real Time (SMRT) sequencing system. Current PacBio sequencing averages ∼10-kb read lengths with consensus sequencing error rates approaching that of NGS. In 2014, Oxford Nanopore Technologies (ONT) introduced the MinION nanopore sequencer. The MinION directly connects to a laptop or desktop PC via a USB3 port, weighs ∼90 g, and costs are for consumable reagents only. The MinION works on the principle of nanopore strand sequencing in real time (Deamer et al. 2016; Jain et al. 2016) and results in sequence read lengths from several hundred bases to hundreds of thousands of bases. To date, most studies have used the MinION to sequence small genomes or to partially survey larger genomes to assess chromosomal structure and copy-number variations (Goodwin et al. 2015; Loman et al. 2015; Norris et al. 2016; Wei and Williams 2016). The long read lengths combined with recent improvements in performance and the development of software to assemble genomes from long sequence reads (Koren et al. 2017) make MinION sequencing a viable option for whole-genome sequencing of complex metazoan genomes.

The *Caenorhabditis elegans* genome was the first metazoan genome to be completely sequenced (The *C. elegans* Sequencing Consortium 1998) and is an excellent model genome for assessing new whole-genome sequencing technologies. The ∼100-Mb *C. elegans* genome is organized into six chromosomes and a mitochondrial genome, and it is complex, with many different types of local repeat and dispersed sequences, the most common dispersed repeats being transposable elements, which constitute ∼12% of the genome (Bessereau 2006). *C. elegans* transposons range in size from 1 to 3 kb and can confound genomic assemblies, as the transposons are longer than NGS and Sanger sequencing reads, resulting in ambiguous mapping positions. Even more problematic for genome assembly are local repeats that range from short repetitive sequences, such as homopolymeric G tracts (Zhao et al. 2007), to large tandem repeats spanning tens of kilobases. Although much effort was invested in the reference genome, it was generated before the availability of longer single molecule sequence reads, and a recent study reports that some repeat regions may be truncated in the reference genome (Li et al. 2015). Here, we report the MinION-derived sequencing and de novo assembly of two *C. elegans* genomes, a complete *C. elegans* reference genome and a strain with two complex chromosomal rearrangements.

## Results

### MinION sequencing of a wild-type *C. elegans* genome

**Figure 1.**
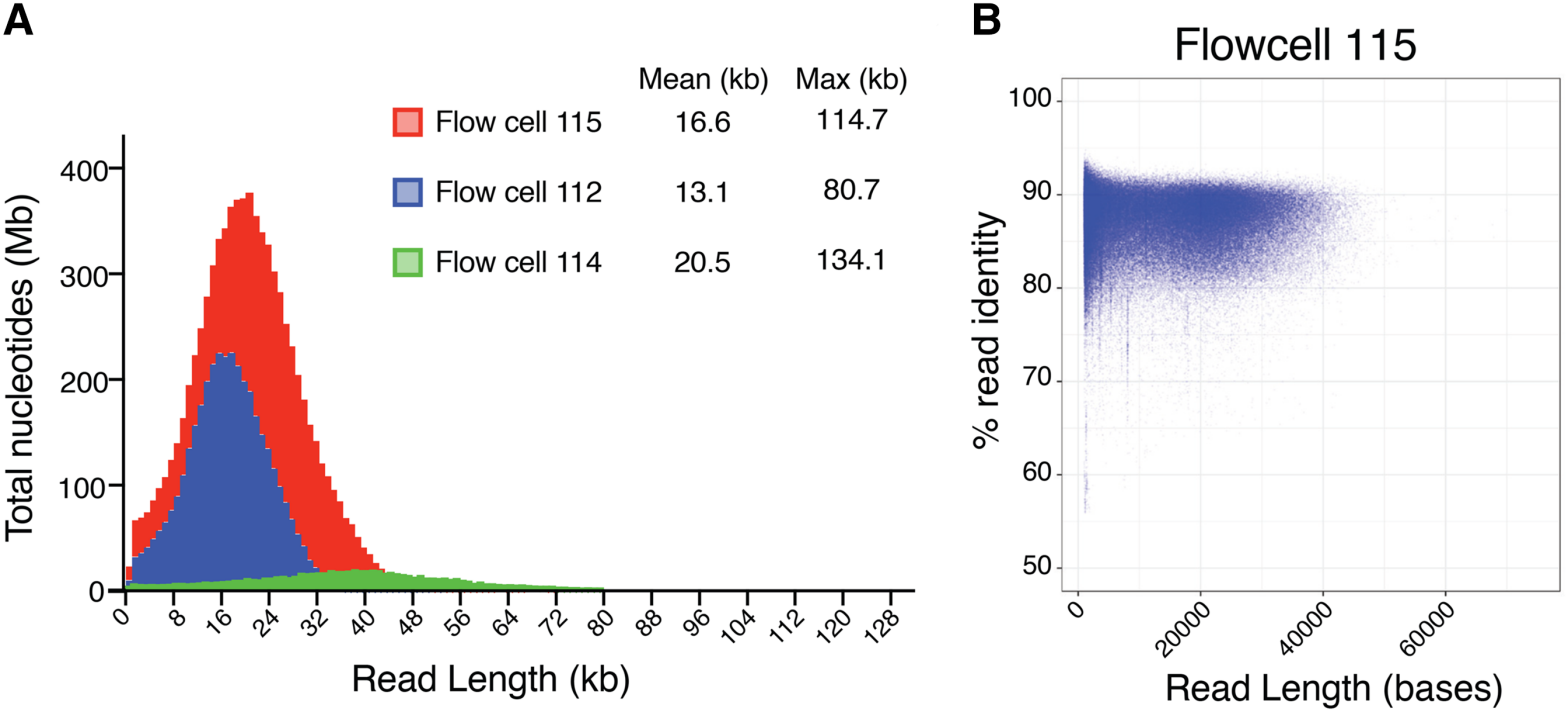
Summary of MinION sequence reads of the *C. elegans* VC2010 wild-type strain genome. (*A*) Histograph of read lengths from flow cells 112, 114, 115. (*B*) Plot of % read identity aligned to the *C. elegans* reference genome vs. read length from flow cell 115. Mean % identities ranged from 85.90% to 86.35% for the three flow cells (Supplemental Fig. S1).

To assess the feasibility of using the MinION platform to generate high quality de novo whole-genome assemblies of large complex genomes, we examined the *C. elegans* wild-type strain VC2010 (Flibotte et al. 2010). Three libraries were constructed from sheared VC2010 genomic DNA (15–20 kb) (Table 1). Each library was sequenced separately on an individual MinION flow cell using custom tuning script modifications inserted into the standard MinKNOW software, and bases were called using the Albacore basecaller from Oxford Nanopore Technologies (see Methods). Individual flow cells resulted in a combined 1,116,324 reads containing 13,485,848,450 bases. In order to improve sequence quality, reads were filtered by size (reads > 1 kb) and quality (Albacore-generated score of q10). Details of the individual MinION sequencing runs can be found in Figure 1A and Table 1 (Supplemental Table S1). Notably, the mean flow cell read lengths ranged from ∼13 kb to ∼ 20 kb, with a maximum read length of ∼134 kb. Nanopore read accuracy was measured by alignment to the *C. elegans* reference genome. Reads from individual flows cells were compared to the reference genome and showed ∼86% nucleotide identity (Fig. 1B; Supplemental Fig. S1A). Mapping all filtered reads to the *C. elegans* reference genome resulted in ∼60-fold coverage (Supplemental Fig. S1B).

**Table 1.**
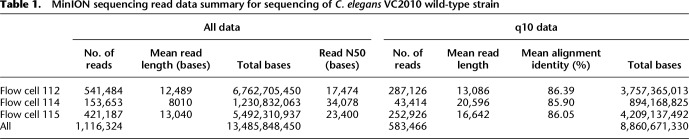
MinION sequencing read data summary for sequencing of *C. elegans* VC2010 wild-type strain

### De novo assembly of the *C. elegans* genome from MinION sequencing

**Figure 2.**
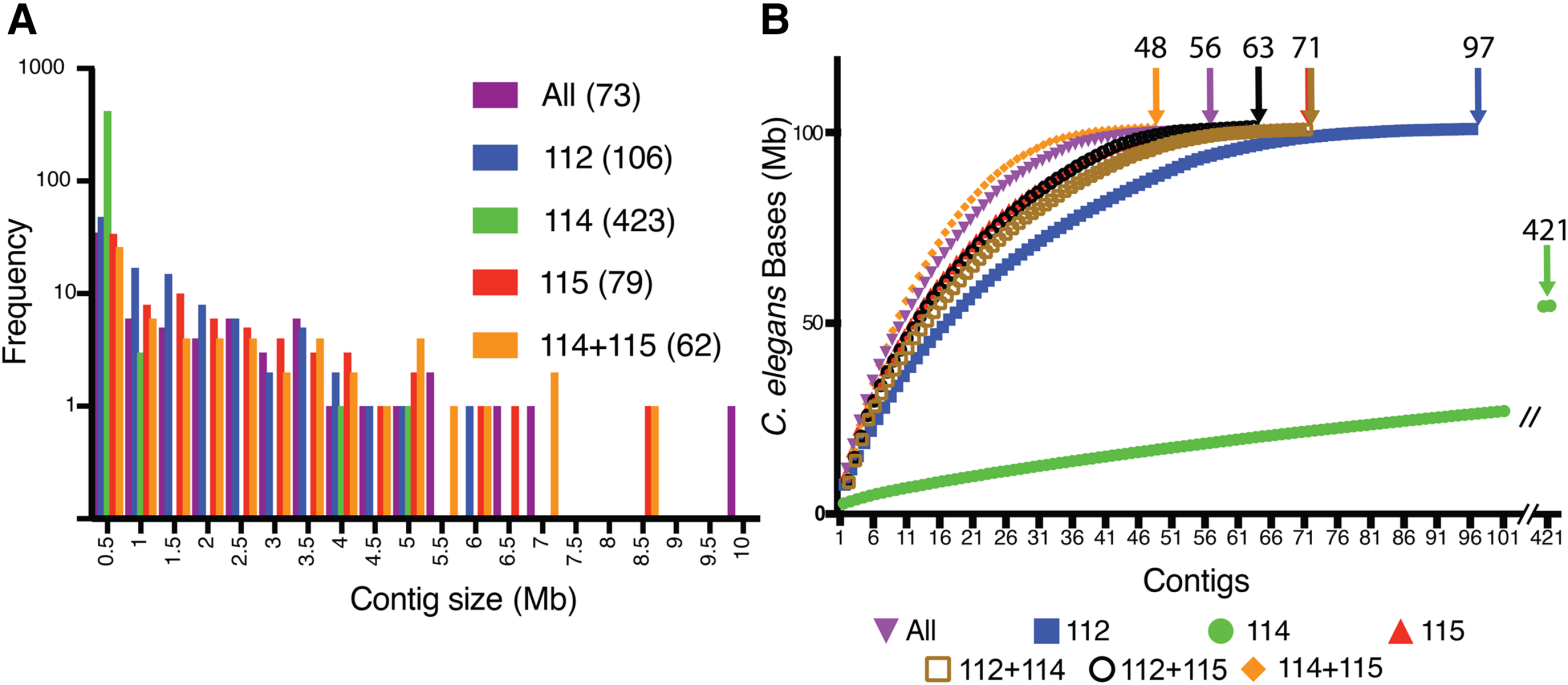
De novo *C. elegans* genome assembly from MinION-generated long reads. Comparison of Canu-generated genome assemblies for individual and combined flow cell data. (*A*) Distribution of contig sizes for different assemblies. Number in parentheses refers to total contigs in the assembly. (*B*) Plot of contig coverage of the reference genome from assemblies produced from the individual and combined flow cells. Arrows denote the number of contigs that align to the *C. elegans* reference genome.

Canu (Koren et al. 2017), a genome assembler designed for high-noise, single molecule sequencing, was used to assemble contigs. Canu assemblies were performed on wild-type *C. elegans* VC2010 Albacore 1.0.1-derived sequence data: one for each individual flow cell, three using pairwise combinations of flow cell data, and one using data from all flow cells are discussed below (Table 2; Supplemental Tables S2, S3). Assembly using q10 reads from all three flow cells, representing ∼60-fold coverage, resulted in 73 contigs, 56 of which had homology to the existing reference genome. The largest contig was >9.9 Mb, and ∼50% of the reference genome was contained in the 10 largest contigs (Fig. 2A). Assemblies using reads from the individual flow cells 112 (3.7 Gb total sequence) and 115 (4.2 Gb total sequence) were similar to the assembly using reads from the combined flow cells. Flow cell 114 had significantly fewer reads (0.9 Gb total sequence) and resulted in a highly discontinuous assembly*.* The most contiguous assembly was not generated from all combined flow cell data; rather, combined flow cells 114 and 115 provided the most contiguous assembly (62 contigs, 48 with *C. elegans* homology). Pooling data from flow cells 114 and 115 resulted in the highest average (and median) read lengths (17 kb) and the highest read length N50s (25 kb) for any combination of flow cells. This suggested that the optimized combination of these parameters yields the best assemblies. To test this hypothesis, Canu assemblies were generated from all reads filtered by size (>10 kb, 7.9 Gb total sequence, >15 kb, 6.7 Gb total sequence, >20 kb, 4.9 Gb total sequence). The >15 kb filter data set resulted in the most contiguous assembly (60 contigs) compared to the >10 kb (65 contigs) and the >20 kb (63 contigs) data sets (Supplemental Table S3). The genome assemblies are illustrated in Figure 2B, which shows the cumulative coverage of the reference genome using contigs with worm homology for each data set.

**Table 2.**
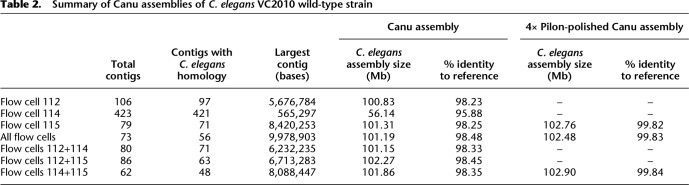
Summary of Canu assemblies of *C. elegans* VC2010 wild-type strain

**Figure 3.**
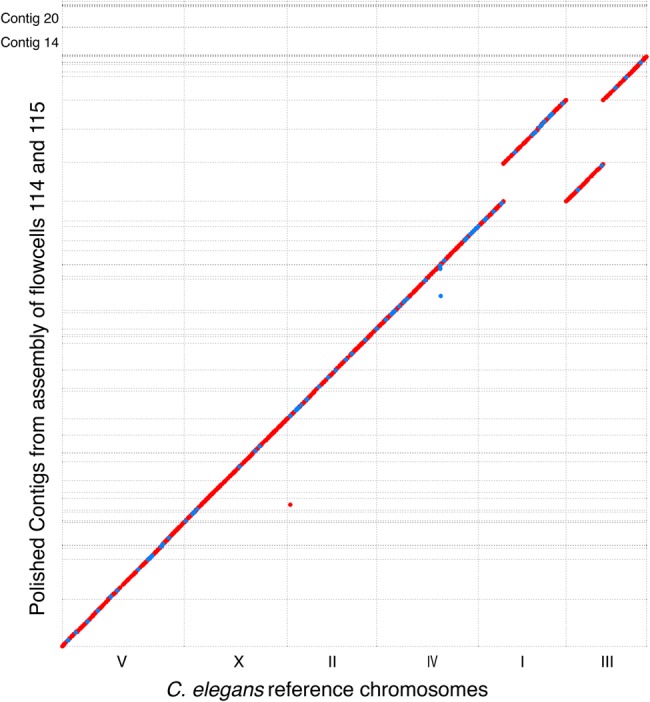
MinION produces high contiguity *C. elegans* genome assembly. MUMmer alignment plot of contigs from assembly of combined data from flow cells 114 and 115 against the *C. elegans* reference genome. The *C. elegans* chromosomes are arranged by size along the *x*-axis and 114+115 contigs along the *y*-axis. Forward strand matches are in red and reverse strand matches are in blue. Note that the large bacterial contigs 14 and 20 do not align to the *C. elegans* reference genome.

Genome assembly utilizing Canu resulted in consensus contig sequence identities of ∼98% when comparing to the reference genome compared to individual read identities of ∼86%. The contigs were further corrected with Illumina short reads using four rounds of Pilon (Walker et al. 2014) polishing, resulting in both correction of mismatches and indels in the MinION data, increasing sequence identity to 99.8% and adding ∼1Mb of sequence (Table 2; Supplemental Table S2). The alignment of the polished contigs from the flow cell 114 and flow cell 115 data was plotted against the reference genome using MUMmer (Fig. 3; Kurtz et al. 2004). The MUMmer alignment of both assemblies demonstrated excellent agreement with the reference sequence (Supplemental Table S2A,B).

### Metagenomic assembly of two bacterial genomes from the *C. elegans* DNA sample

Two large contigs, contig14 (4.8 Mb) and contig20 (3.5 Mb) from the 114+115 assembly, did not align to the *C. elegans* reference genome (Fig. 3). Contig14 appears to be a complete genome of a novel strain of *Stenotrophomonas maltophilia*, most similar to strain R551-3 (99% identity) (Supplemental Fig. S2), and contig20 appears to be a novel bacterial genome with similarity to *Brucella suis* and *Ochrobactrum anthropi* based on RAST analysis (Aziz et al. 2008). Both *S. maltophilia* and *Ochrobactrum* are soil bacteria known to be part of the *C. elegans* native microbiome and are important human nosocomial pathogens. It is likely that both were contaminants in the nematode cultures that were prepared for sequencing. Previously, a *S. maltophilia* genome was derived from *Caenorhabditis remanei* tissue (Fierst et al. 2017), resulting in two contigs. The current MinION-derived assembly is more complete, consisting of a single contig. These data demonstrate the utility of long reads to assemble complete genomes from mixed samples and we hypothesize could be used to survey the microbiome of *C. elegans* isolates.

### MinION-derived assemblies identify sequences missing in the reference genome

**Figure 4.**
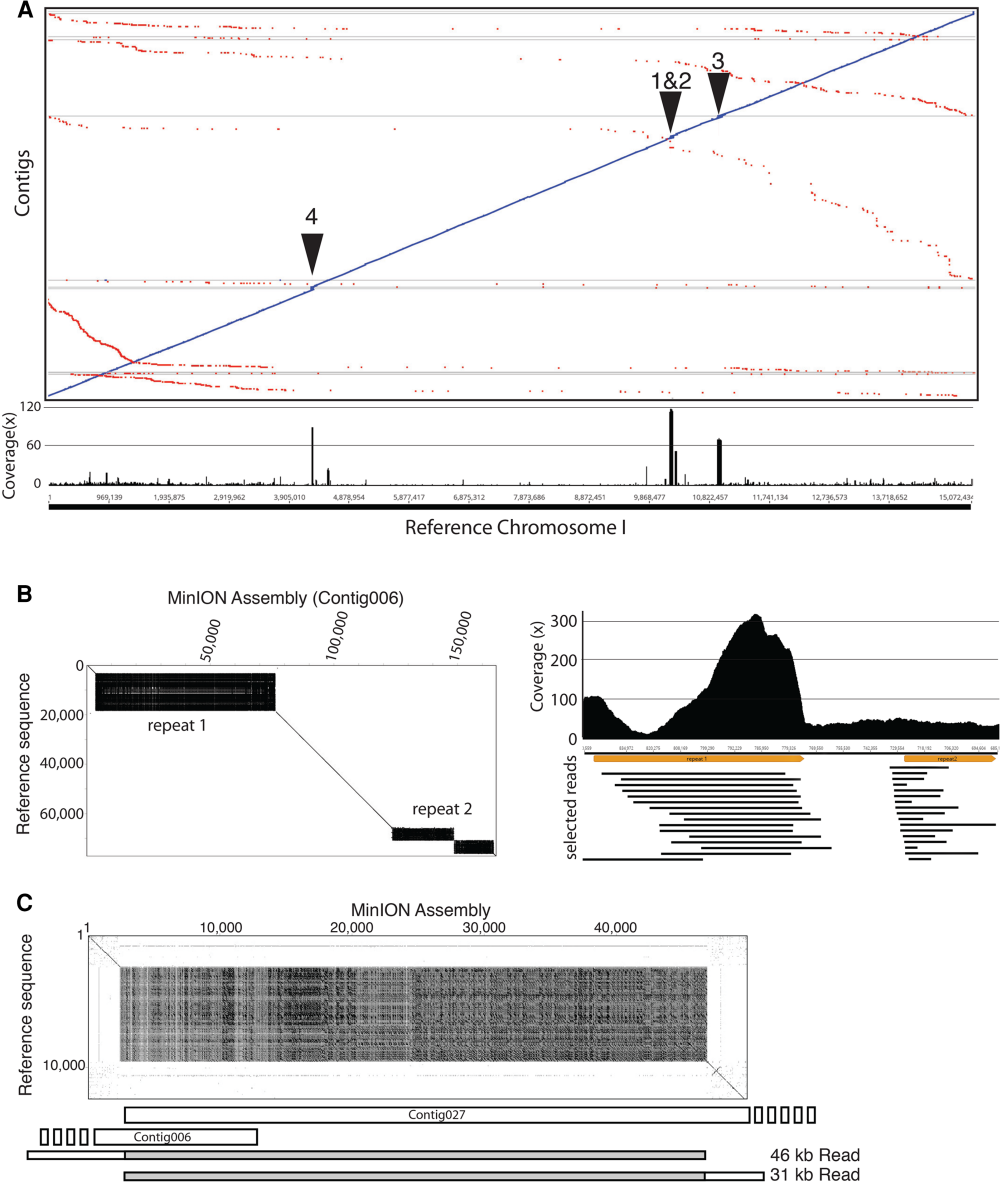
MinION sequencing and assembly reveals genome expansion of *C. elegans* repeat regions. (*A*) LASTZ alignment of contigs to the *C. elegans* reference Chromosome I. *Below* is a plot of coverage of the ‘All data’ assembly contig sequences against the reference. Note the high coverage areas (indicated as 1–4) that correlate with repeat regions. (*B*) (*Left*) Dot plot of the MinION-derived contig containing repeats 1 and 2 against the reference sequence. (*Right*) Plot of sequence read coverage (reads > 5 kb) in the repeat regions. Selected reads spanning the expanded repeats are shown *below*. Note the high coverage of repeat 1, which suggests that this repeat is even larger than predicted in the contig shown. Repeat 2 read coverage, which is similar to the average read coverage across the genome, suggests that this repeat expansion is correct. (*C*) Dot plot of the end termini of contig006 and contig027 against the reference sequence demonstrating the expansion of repeat 3. Two of the longest reads mapping to this repeat are shown *below* with the repeat highlighted in gray.

The MinION-derived *C. elegans* genome assemblies were substantially larger than the reference sequence when aligned (∼102.9 Mb compared to 100.3 Mb) (Table 2), and alignment did not identify any large unaligned regions that would account for the discrepancy. Plotting coverage depth of contig sequence generated from all q10 reads against the reference genome using the LASTZ aligner (Harris 2007) revealed that much of the increased genome size was due to expansions of repeat regions present in the MinION-derived assemblies relative to the reference genome. The LASTZ alignment of assembly contigs against the reference genome and coverage for Chromosome I are shown as an example in Figure 4A. Expanded repeat regions were found contained within contigs and at the ends of contigs. The four largest potential expansions on Chromosome I, two internal to a contig and two on contig ends, were investigated further. The two expanded repeats within a contig (repeat 1 and 2), a 112-copy repeat of a 136-mer and a 17-copy repeat of a 231-mer, are located between 10,202,622 and 10,277,710 of Chromosome I. These tandem repeats are approximately fivefold larger in the MinION-derived genome assembly, more than doubling the size of this region from ∼73 kb to ∼161 kb. The expansions are unlikely to be the result of Canu assembly errors as they were identified on multiple independent long reads (Fig. 4B). On Chromosome I, contigs 6 and 27 share an end-terminal tandem repeat (repeat 3) and can be ordered and oriented with respect to each other (Fig. 4C). However, no single read completely spanned the expanded repeat, so the actual repetitive region may be even larger. Similarly, contigs 299, 300, and 302 can be ordered based upon sequence homology of repeat 4 at their contig ends.

### MinION reads elucidated the structure of complex repetitive chromosome rearrangements

In addition to de novo genome assembly, long sequencing reads can also facilitate the delineation of complex chromosomal rearrangements that contain multiple breakpoints, duplications, deletions, and repeated sequences. To assess the feasibility of using MinION sequencing to resolve complex rearrangements, a *C. elegans* strain containing two homozygous complex rearrangements, *him-9(e1487)* II; *unc-119(ed3) ruIs32[pie-1p::GFP::H2B + unc-119(+)]* III, was constructed. The *him-9(e1487)* mutation was induced by acetaldehyde mutagenesis (Hodgkin et al. 1979) and is a complex duplication and insertion event that disrupts the predicted *C. elegans XPF* ortholog *xpf-1* (Youds et al. 2006). Previous analysis of *him-9(e1487)* from oligo array hybridization, reverse transcriptase PCR, and inverse-PCR experiments suggested that *him-9(e1487)* was a duplication of ∼20 kb of sequence from the *mab-3* region that had been inserted into the second intron of the *xpf-1* gene (NJ O'Neil, unpubl.). However, the complex nature of the mutation hindered attempts to determine the exact structure of the rearrangement. *ruIs32* is a low-copy-number insertion that was generated by biolistic transformation of the plasmid pAZ132 [*Ppie-1::GFP::H2B::pie-1*] and a plasmid containing *unc-119*[*unc-119(+)*] into an *unc-119(ed3)* mutant (Praitis et al. 2001). Genomic DNA was prepared from the *him-9(e1487)* II; *ruIs32* III strain and sequenced using MinION.

Sequencing runs from six MinION flow cells using older R9.0 (4) and R9.3 (2) pore flow cell types were performed (see Methods). This resulted in 1.1 M individual reads up to 123,159 bases in length (mean = 4801) and containing 5.33 Gb of 1D bases. An additional 1 Gb of 2D sequence was generated from the paired template and complements 1D reads produced from the R9.0 flow cells using the SQK-NSK007 2D chemistry and Metrichor basecaller. Mapping reads to the reference genome with the BWA-MEM aligner (Li 2013) resulted in ∼45-fold coverage (Supplemental Fig. S3). A Canu assembly was performed using all “pass” reads to generate an assembly containing 236 contigs, which were polished using Pilon and Illumina sequence data (Supplemental Tables S2C, S4). Pilon-polished contigs ranged in length from 4160 to 5,740,216 bases, with 100 of the contigs (representing 102,308,633 bases) having significant homology to the *C. elegans* reference genome. The mean length for the 100 *C. elegans* matching contigs was 1.1 Mb, and they were aligned to the WBcel235 release of the *C. elegans* reference genome using MUMmer (Supplemental Fig. S4; Supplemental Table S3C). The alignment of contigs to the six chromosomes and the *C. elegans* mitochondrial genome covered >99% of the reference genome, with most contigs having a >99% identity match to the reference genome. There were no large gaps in coverage of the reference genome. Consistent with the MinION-derived VC2010 genome, the *him-9; ruIs32* genome was ∼2% larger than the reference genome.

**Figure 5.**
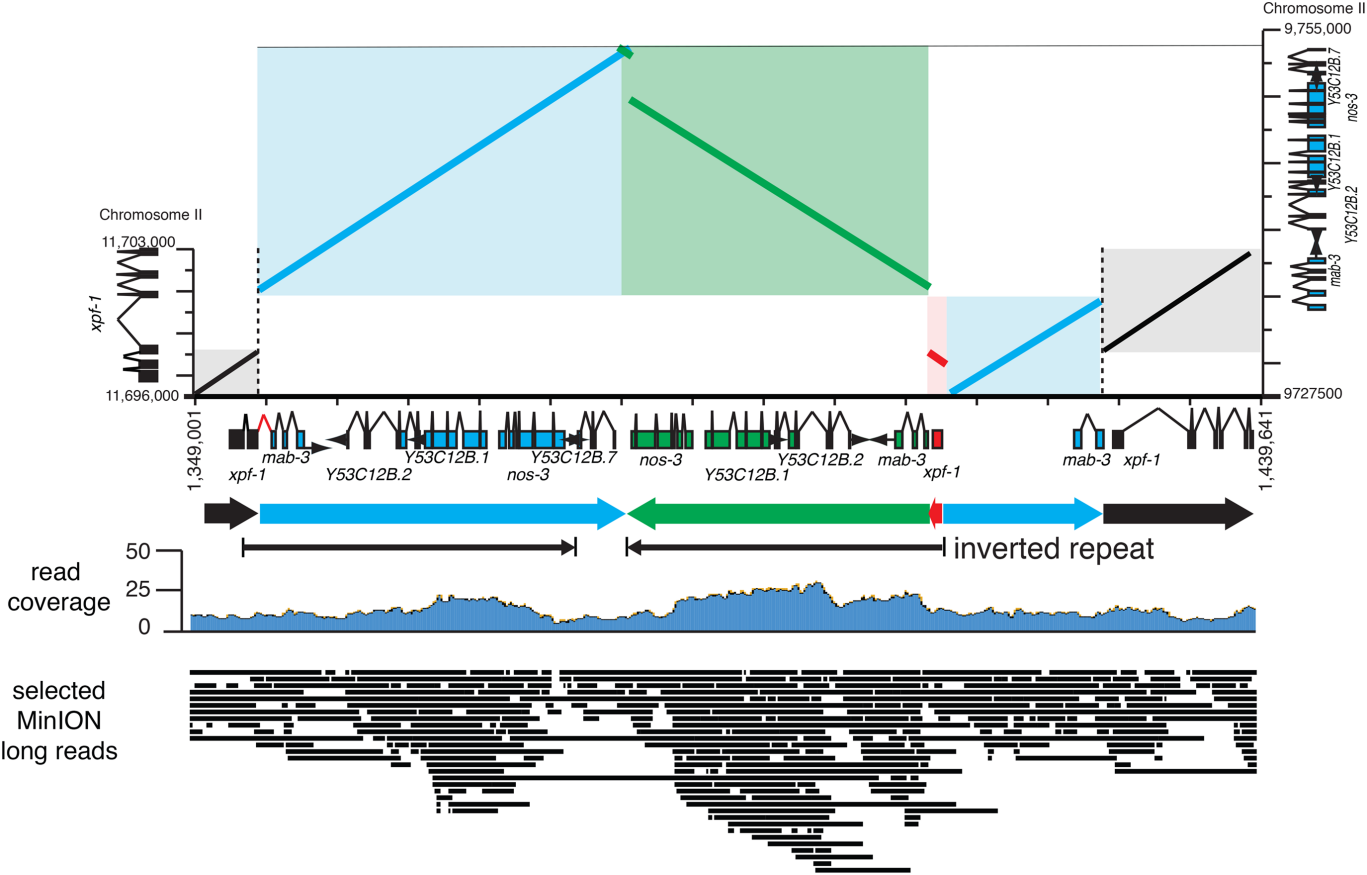
Delineation of complex genome rearrangements. Schematic of the *xpf-1(e1487)* complex mutation in contig017. The *left y*-axis shows the wild-type *xpf-1* gene structure and the *right y*-axis shows the *mab-3* region. The mutation is a duplication and insertion of ∼20 kb of the *mab-3* region in two segments into the second intron of *xpf-1* (blue). The larger segment of the inserted region along with the flanking *xpf-1* exon 2 has been duplicated creating an inverted repeat (green and red). Shown *below* is read coverage and selected MinION reads mapping to the region spanning the various breakpoints.

The Canu assembly of MinION reads included a single contig (contig017) containing the entire *him-9(e1487)* rearrangement. In addition to the predicted ∼20-kb *mab-3* duplicated region*,* the insertion was found to also include an inverted repeat of part of the *mab-3* duplication and the second exon of *xpf-1,* resulting in a much larger, more complex insertion than expected (Fig. 5).

**Figure 6.**
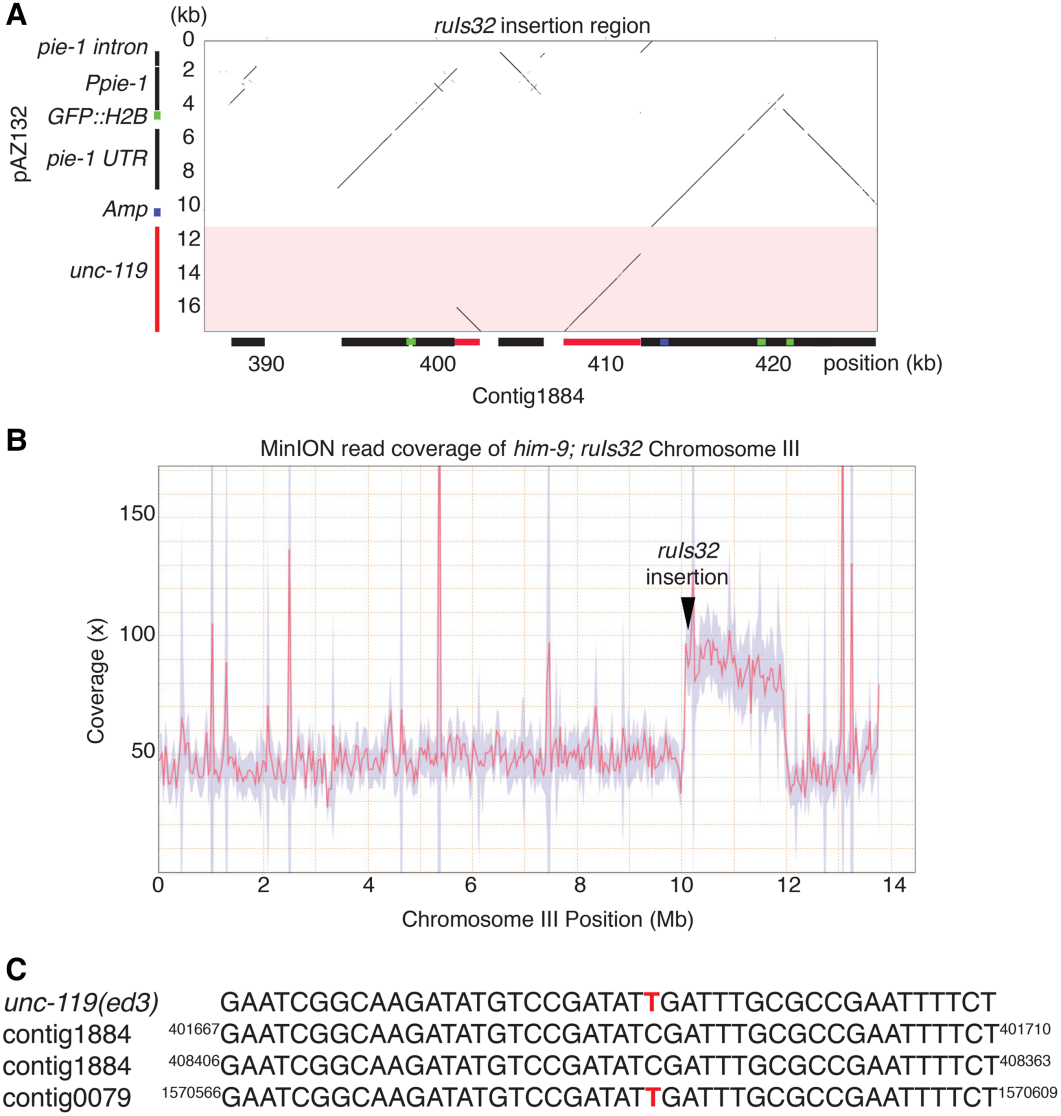
Delineation of an exogenous plasmid DNA array integration and duplication event. (*A*) Dot plot of the *ruIs32* insertion in contig1884 against the pAZ132 plasmid and *unc-119* gene (red) that were integrated by biolistic transformation. The *y*-axis shows the pAZ132 plasmid structure and the *unc-119* gene structure. The insertion contains three copies of GFP::H2B and two partial copies of *unc-119*. (*B*) MinION read coverage for Chromosome III. Note the ∼2-Mb duplication in the region where *ruIs32* has been integrated. Red line is coverage and purple shading is coverage deviation. (*C*) MinION-generated sequence identified the two wild-type copies of *unc-119* inserted with *ruIs32* and the *unc-119(ed3)* mutation in contig0079. Superscript numbers refer to position within the contig.

The biolistic-mediated insertion was found to be located on contig1884, which aligned to the right arm of Chromosome III, consistent with the published location for *ruIs32* (WormBase). From the MinION read assembly, it appears that the insertion contains three copies of the *Ppie-1::GFP::H2B::pie-*1 transgene and two copies of the ampicillin gene from the plasmid and two partial copies of the *unc-119(+)* gene from the *unc-119* transgene. The structure of the insertion is complex, with the *unc-119(+)* sequence interspersed within the plasmid sequence, suggesting that a complex integration event occurred (Fig. 6A). Interestingly, the integration event also appears concomitantly with a large duplication of ∼2 Mb of DNA (Chr III: 10,062,096–11,973,739) from the region near the insertion site (Fig. 6B). Given the position of the insertion, the wild-type *unc-119* transgene should be genetically linked to the *unc-119(ed3)* mutation and not be lost through outcrossing*.* Indeed, both the *unc-119(ed3)* mutation and two wild-type transgenic alleles could be discerned from the MinION contigs (Fig. 6C). From these data, it is evident that the long reads from MinION can be used to resolve complex genome rearrangements, and we predict that this strategy will prove useful for other types of analyses aimed at characterizing rearrangements associated with tumors.

## Discussion

Advances in long-read sequencing have opened new avenues for genome analysis. The relatively short sequencing reads produced by NGS allow for effective and cost-efficient resequencing of less complex, nonrepetitive regions in well-characterized genomes. Long sequence reads can span repetitive regions and facilitate contiguous assemblies of large contigs. Similarly, long reads can resolve complex chromosomal alterations such as those observed in tumor genomes, as well as provide positional context needed to assemble individual unique genomes from mixed samples containing many different genomes. The major challenges limiting the use of long-read sequencing technology have been throughput, accuracy, and cost. Here, we present the de novo assembly of MinION sequencing reads generating a remarkably complete wild-type genome in 48 contigs covering >99% of the reference genome. Notably, the MinION assembly improved the reference genome by expanding repetitive regions that could not be resolved by previous sequencing strategies and added more than 2 Mb of sequence. MinION long reads were also used to delineate the complex chromosomal rearrangements in a mutant strain. These data demonstrate that MinION is a useful platform for generating and characterizing complex metazoan genomes and that accuracy from the assembled MinION generated data can be further improved by ‘polishing’ with higher accuracy short-read data.

### Assemblies are most impacted by read length

The MinION-derived genome assemblies demonstrated that both the direct sequencing and de novo assembly of a large, low percent-GC (35.44%), complex metazoan genome can be accomplished using MinION sequencing reads alone. Near-complete *C. elegans* genome assemblies (>99% coverage) consisting of large contigs could be assembled from single MinION flow cells. These assemblies are significantly more contiguous than those generated by Illumina Synthetic Long-Read Sequencing or by PacBio sequencing for the *C. elegans* genome. MinION-derived assemblies resulted in an assembly with an N50 contig size of 3.99 Mb compared to an N50 of 86 kb for Synthetic Long-Read Sequencing (Li et al. 2015) and an N50 of 1.6 Mb for PacBio sequencing (https://github.com/PacificBiosciences/DevNet/wiki/ C.-elegans-data-set). Combining flow cell data improved the assemblies. Interestingly, the most contiguous genome assembly did not come from the combined largest data set (8.9 Gb total sequence, 15 kb mean read length, N50 21 kb) but from flow cells 115 (4.2 Gb total sequence, 16.6 kb mean read length, N50 24 kb) and 114, which contained fewer but longer reads (0.9 Gb total sequence, 20.6 kb mean read length, N50 38 kb). Comparisons of assemblies from data sets filtered by read length further support the hypothesis that adding more reads can reduce assembly quality if those reads decrease the average reads length. Contig breakpoints were located most often in regions containing repetitive elements that were larger than the longest read lengths. By generating and including reads longer than these repeat structures, even these repetitive regions should be spanned, resulting in a more contiguous genome. It is clear from these assemblies that deeper sequencing of DNA libraries of similar sized fragments would not have resolved the contig breaks. Rather, we suggest that these breakpoints could be spanned by preparing genomic DNA to favor the formation of larger fragments.

### MinION sequence improves the *C. elegans* reference genome by expanding repeat regions

The MinION-generated genome assemblies were considerably larger (>2 MB) than the *C. elegans* reference genome. This was due to more accurate assembly of large repetitive elements in the *C. elegans* genome, resulting in an improved genomic reference. It has recently been reported that the extent of some repetitive elements in the reference genome are truncated (Li et al. 2015). The MinION-generated genome presented here expands repetitive elements throughout the genome, adding more than 2 Mb to the reference genome. These are unlikely to be strain-specific expansions as the same expansions were observed in the sequencing of the *him-9;ruIs32* strain, although the expansions were smaller due to the fact that the fragment length of the *him-9* library was considerably shorter than the fragment length in the VC2010 libraries. We hypothesize that the reference genome could be further improved by sequencing ultra-long DNA fragments (100–1000 kb), which are being made possible by the development of DNA extraction techniques and library construction protocols that reduce shearing of genomic DNA (Jain et al. 2017). Such ultra-long-read sequencing could truly complete the *C. elegans* reference genome by spanning the extremely large repetitive elements such as the rDNA cluster.

### MinION sequencing can resolve complex rearrangements

Chromosome rearrangements are common in tumors, and the identification of rearrangements is important for tumor characterization and treatment. Detecting chromosome rearrangements such as duplications, deletions, and translocations poses challenges for sequencing-based approaches. NGS can detect copy-number changes and identify potential breakpoints. However, complex rearrangements with multiple breakpoints and duplications can prove difficult to reassemble from NGS reads. The long MinION reads provided context for the delineation of complex chromosomal rearrangements. MinION long reads offer the opportunity to identify genome rearrangements in tumors, including the highly complex chromothripsis events which can result in thousands of clustered localized chromosomal rearrangements (Leibowitz et al. 2015).

Our sequencing and assembly of the *C. elegans* genome demonstrates the advancing capabilities of the Oxford Nanopore Technologies sequencing platform. Throughput and accuracy of MinION continues to improve and is approaching 10–15 Gb of sequence per flow cell, which is sufficient sequence to assemble the 100-Mb *C. elegans* genome to a high continuity. The long-read capabilities of MinION nanopore sequencing further facilitate unambiguous assembly of chromosome structure, thereby eliminating the need for physical mapping, and can delineate complex chromosome rearrangements and extrachromosomal DNA elements. These properties should allow for sequencing of new genomes or tumor genomes with structural chromosome changes or micronuclei in addition to improving existing reference genomes by more accurately sequencing repetitive elements. Combining MinION and Illumina sequencing currently can delineate the structure of novel genomes with higher base-level certainty.

## Methods

### Nematode culture and DNA extraction

Nematodes were cultured as previously described (Brenner 1974). The *him-9(e1487)* II; *unc-119(ed3) ruIs32[pie-1p::GFP::H2B + unc-119(+)]* III strain was constructed by mating CB1487 *him-9(e1487)* males to AZ212 *unc-119*(*ed3*) *ruIs32* III hermaphrodites. F1 heterozygotes were selfed and *him-9; ruIs32* homozygotes isolated. Worms were grown to starvation for sequencing on NGM plates and harvested by washing with M9 buffer and pelleted in 15-mL centrifuge tubes. Buffer was removed by two washes with sterile distilled water, centrifugation, and aspiration. The worm pellet was resuspended in 300 µL of lysis buffer (200 mM NaCl, 100 mM Tris-HCl pH 8.5, 50 mM EDTA pH 8.0, 0.5% SDS, 0.1 mg/mL proteinase K) and frozen at −80°C. Frozen pellets were incubated at 60°C for 1–3 h followed by 95°C for 20 min. RNase A (0.1 mg/mL) was added, and lysed worms were incubated at 37°C for 1 h. DNA was prepared by standard phenol/chloroform extraction, and DNA was resuspended in 10 mM Tris pH 8.0.

### Illumina library preparation and sequencing

The DNA library for VC2010 N2 was constructed by Novogene and sequenced with the Illumina HiSeq 2000 platform. In brief, a total amount of 1.0 µg DNA was used as input material. A sequencing library was generated using the TruSeq Nano DNA HT Sample Preparation kit (Illumina) following the manufacturer's recommendations, and index codes were added to attribute sequences. The genomic DNA was randomly fragmented to a size of 350 bp by Covaris cracker, then DNA fragments were end-polished, A-tailed, and ligated with the full-length adapter for Illumina sequencing with further PCR amplification. Finally, PCR products were purified (AMPure XP system), and libraries were analyzed for size distribution by an Agilent2100 Bioanalyzer and quantified using real-time PCR. The qualified libraries were sequenced by a HiSeq sequencer. The DNA library for the *him-9; ruIs32* strain was constructed using a Nextera DNA library preparation kit (Illumina) and sequenced with a 600-cycle MiSeq v3 reagent kit on an Illumina MiSeq platform.

### Library preparation and MinION sequencing

MinION flow cells 84 to 90 were run using libraries prepared with the SQK-NSK007 Nanopore Sequencing Kit R9 version. Libraries for flow cells 94 and 95 were prepared using the SQK-RAD001 Rapid Sequencing Kit I R9 version. Libraries for flow cells 112, 114, and 115 were prepared using the SQK-LSK108 1D Ligation Sequencing Kit. The standard protocols from Oxford Nanopore Technologies were used with the following modifications. For SQK-NSK007 libraries, purification of DNA after the FFPE treatment step was done using 0.4× AMPureXP beads. For SQK-LSK108 libraries, DNA fragmentation was performed using 20 passes through 29G (flow cell 112 and flow cell 115) or 26G (flow cell 114) needles in a 200-µL volume before purification using 0.4% AMPureXP beads and then going into the end-repair step of the standard protocol. Prior to adapter ligation, each elution step of the AMPureXP beads was performed using 10 mM Tris pH 8.0 instead of water, at 37°C for 5 min. The starting amounts of gDNA ranged between 0.8 µg and 10.0 µg (see Supplemental Table S1 for details). Priming of individual flow cells with running buffer (500 µL) and sequencing library top-ups (75–150 µL) were performed at times detailed in Supplemental Table S1. Flow cells were run for ∼48 h using device tuning scripts layered onto the standard MinKNOW software. The tuning scripts provide event yield monitoring aimed at maintaining data throughput through initiation of a maximal pore channel assignment/usage strategy and optimal bias-voltage selection via methods outlined below.

### Modified MinION running scripts

After initial start of a MinION sequencer run using the standard ONT sequencing scripts, MinION sequencing control was shifted to a custom MinKNOW MinION script to enhance pore utilization and increase data yields. These custom script modifications adjusted a number of run/flow cell metrics and parameters including:

1. Initiation of a bias-voltage reselection and active pore repopulation into active channels when the hourly event yields fall below a threshold. This threshold was set at 67% of the first hour of each particular sequencing segment and generally ran for 2–5 h per segment.
2. Identifying the bias-voltages that provide the greatest number of active pores by scanning a voltage range (20–30 mV in increments of −10 mV) and using this for active pore channel reassignment. A newly selected bias-voltage acts as the starting point for subsequent scans and provides an active pore “tracking” ability as the required bias-voltage magnitude increases during a run with the electrochemical gradient decay of individual wells. This reassignment also provides access to the full 2048 possible wells repeatedly throughout a run.
3. Selecting a lower magnitude bias-voltage wherein the active pore numbers are within 10% of the peak voltage. Keeping greater pore numbers active using these approaches results in wells/pores running for different periods of time, and the bias-voltage requirement to drive the same current through a pore increases and diverges with use. This is because the electrochemical gradient of active wells/pores decays at a greater rate than that of inactive wells/pores. By using off-peak, lower magnitude bias-voltage selection, a measure of pore population containment or “shepherding” is provided by moving lower magnitude voltage requiring pores into the rest of the population.

More detailed information on these scripts can be found at the Oxford Nanopore Technologies user community. In addition, a patch for all files required to modify MinION running scripts compatible with MinKNOW 1.3.23 only is available (Supplemental Methods).

### Base-calling MinION sequencing reads

Reads generated from the MinION sequencing device for the *him-9(e1487)* strain sequencing were base-called using the cloud-based Metrichor service provided by Oxford Nanopore Technologies. Reads generated for the VC2010 wild-type strain were base-called using the Albacore (0.8.4 [nonhomopolymer calling] and 1.0.1 [homopolymer calling]) software (Oxford Nanopore Technologies). Details of specific versions and comparisons of the effects of Albacore base-calling (0.8.4 [nonhomopolymer calling] and 1.0.1 [homopolymer calling]) on the final sequence identity can be found in Supplemental Table S4 for the VC2010 flow cell 115 assemblies. DNA sequences were extracted from individually called reads using simple Python scripts and combined in a single FASTA file format of a particular strand sequence type and quality. Runs using the SQK-NSK007 (2D) kit generated template and a fraction of complement and 2D sequence from individual reads. SQK-RAD001 and SQK-LSK108 (1D) library runs generated template (1D) reads. Filtering based on a quality metric by Metrichor divided the reads additionally into ‘pass’ and ‘fail’ categories based on a read quality ‘q6’ filter. Some or all of these sequence-containing files for each run were then used for genome assembly as indicated.

### Genome assembly and evaluation

#### Canu nanopore assembly

Selected nanopore data sets were pooled into individual FASTA files as input and reads below a size of 1 kb were filtered out to avoid overlap detection issues. The remaining reads were assembled using Canu (v1.4) (Koren et al. 2017) with the (corMinCoverage=0 cor MaxEvidenceErate=0.22 errorRate=0.045 ′′corMhapOptions=--threshold 0.8 --num- hashes 512 --ordered-sketch-size 1000 --ordered-kmer-size 14′′) command modifications from default.

#### Assembly polishing

The Canu assemblies were corrected using Pilon (Walker et al. 2014) using recommended settings to polish for variants and homopolymers. We did not perform quality filtering on the Illumina data, and all of the short read sequence data were used.

#### Nanopore data and assembly evaluation

Nanopore reads and assemblies were evaluated by alignment against the reference worm genome using BWA-MEM (Li 2013) (in ‘ont2d’ mode). Assembly evaluations were performed using LASTZ (Harris 2007) and MUMmer (Kurtz et al. 2004) alignments of the draft assemblies to the reference.

We evaluated the assembly quality by using MUMmer with recommended settings and the reference worm genome. We compared changes in indels and mismatches for the Canu assemblies before and after Pilon correction (Supplemental Table S4). We evaluated the assemblies based on: (1) the total number of aligned bases in the assembly; (2) the number of mismatches; and (3) the total number of bases contained in indels.

## Data access

Genome assemblies from this study have been submitted to the European Nucleotide Archive (ENA; https://www.ebi.ac.uk/ena) under the following accession numbers: PRJEB22098 (Nanopore reads), PRJEB22099 (Nanopore Assemblies), PRJEB22100 (Illumina reads).

## Supplementary Material

Supplemental Material
