# Supplementary material for: MinION-based long-read sequencing and assembly extends the Caenorhabditis elegans reference genome
Source: Genome Res. 2018 Feb;28(2):266–74. doi: 10.1101/gr.221184.117 (PMC5793790; doi:10.1101/gr.221184.117)
Supplement: Supplemental Material [file supp_28_2_266__index.html]

MinION-based long-read sequencing and assembly extends the Caenorhabditis elegans reference genome — Supplemental Material 

# MinION-based long-read sequencing and assembly extends the *Caenorhabditis elegans* reference genome

## Supplemental Material

- Supplemental\_Fig\_S1.pdf
- Supplemental\_Fig\_S2.pdf
- Supplemental\_Fig\_S3.pdf
- Supplemental\_Fig\_S4.pdf
- Supplemental\_Legends.docx
- Supplemental\_Table\_S1.xlsx
- Supplemental\_Table\_S2.xlsx
- Supplemental\_Table\_S3.docx
- Supplemental\_Table\_S4.xlsx
- Supplemental\_Method.zip
