# Supplementary figures and images for: MinION-based long-read sequencing and assembly extends the Caenorhabditis elegans reference genome
Source: Genome Res. 2018 Feb;28(2):266–74. doi: 10.1101/gr.221184.117 (PMC5793790; doi:10.1101/gr.221184.117)

A

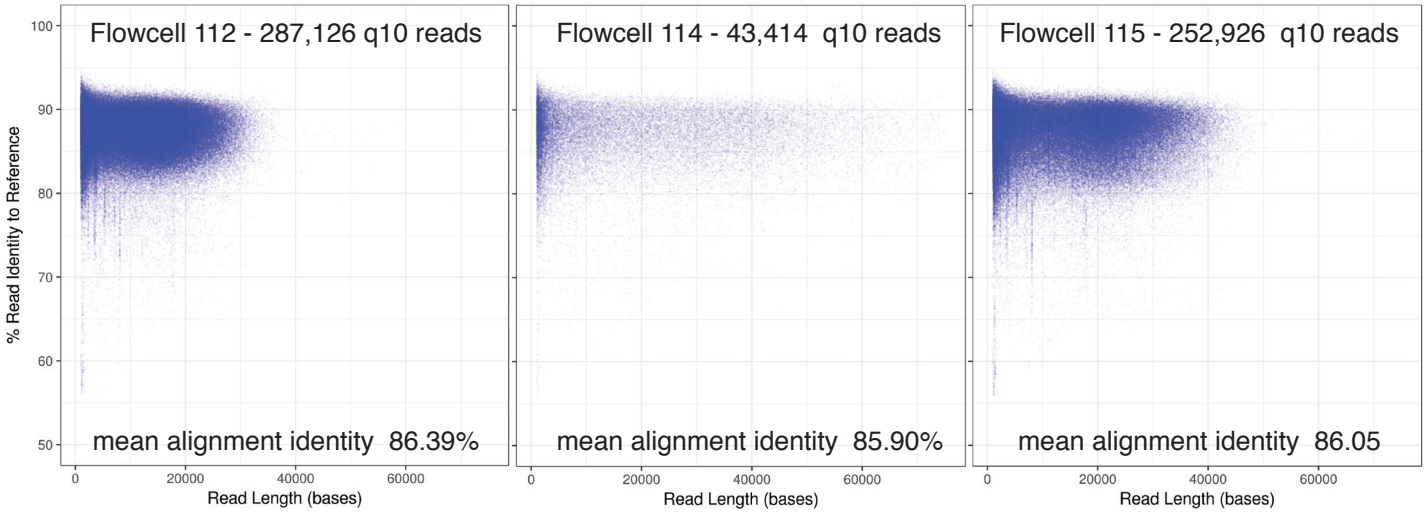

B

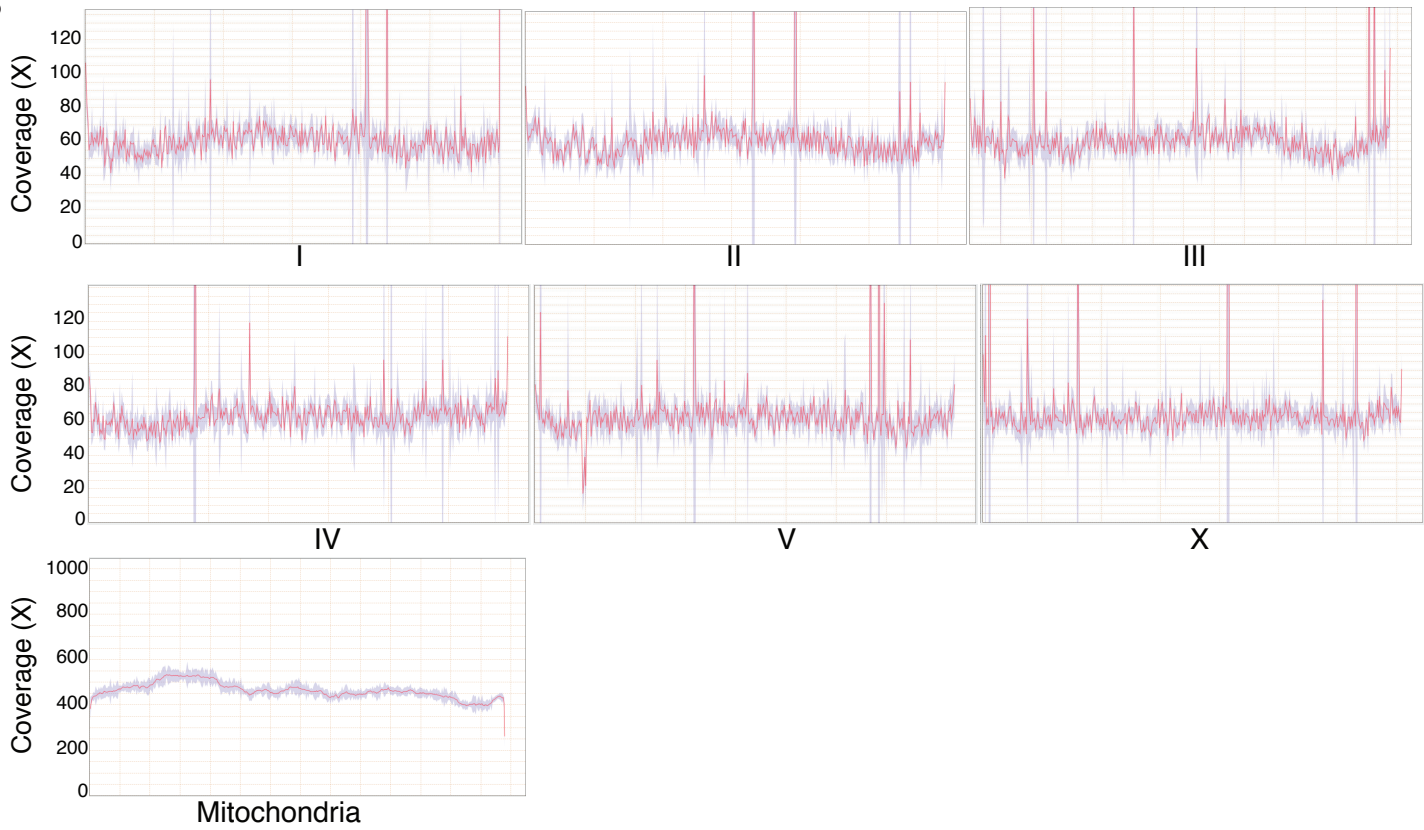

Supplement: Supplemental Material [file supp_gr.221184.117_Supplemental_Fig_S1.pdf]

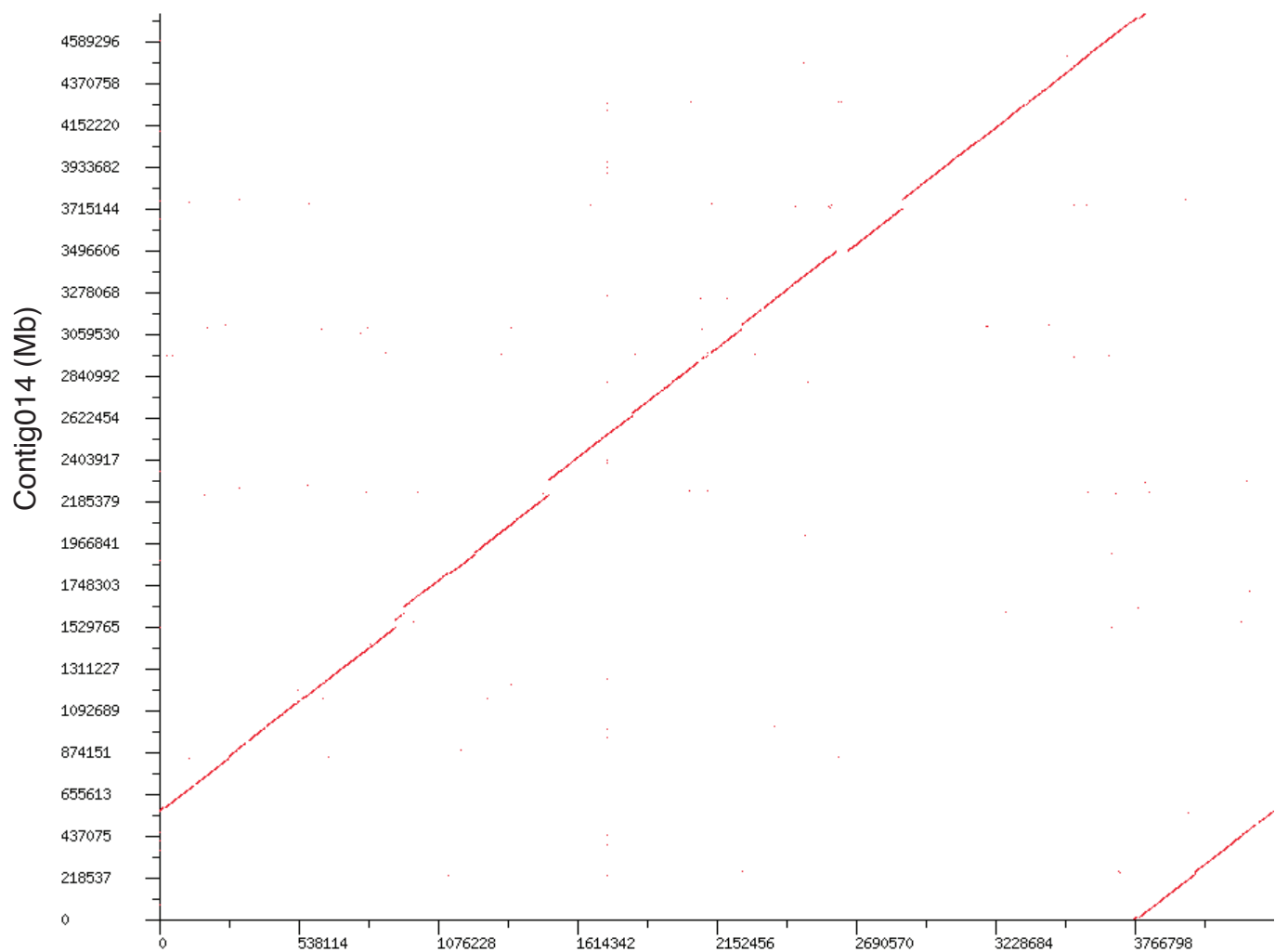

*Stenotrophomonas maltophilia* R551-3

Supplement: Supplemental Material [file supp_gr.221184.117_Supplemental_Fig_S2.pdf]

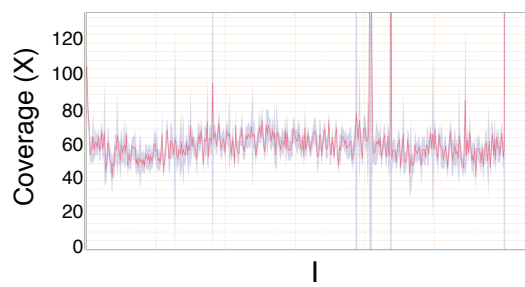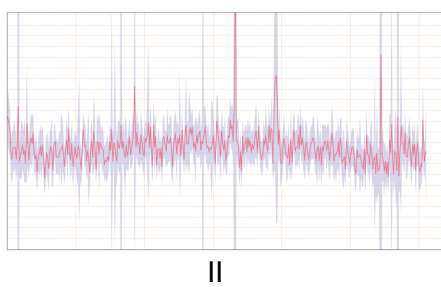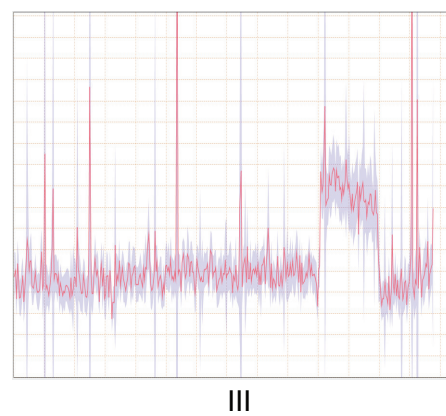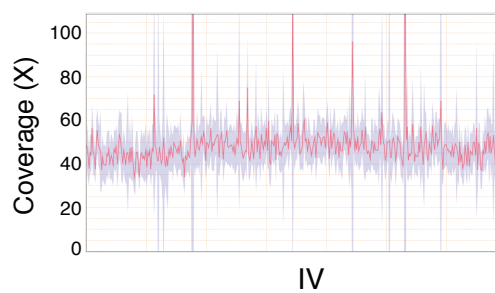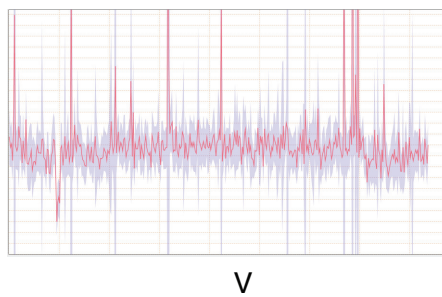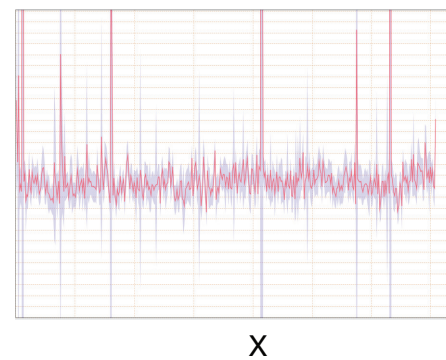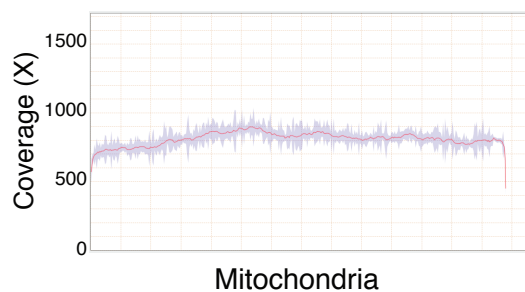

Supplement: Supplemental Material [file supp_gr.221184.117_Supplemental_Fig_S3.pdf]

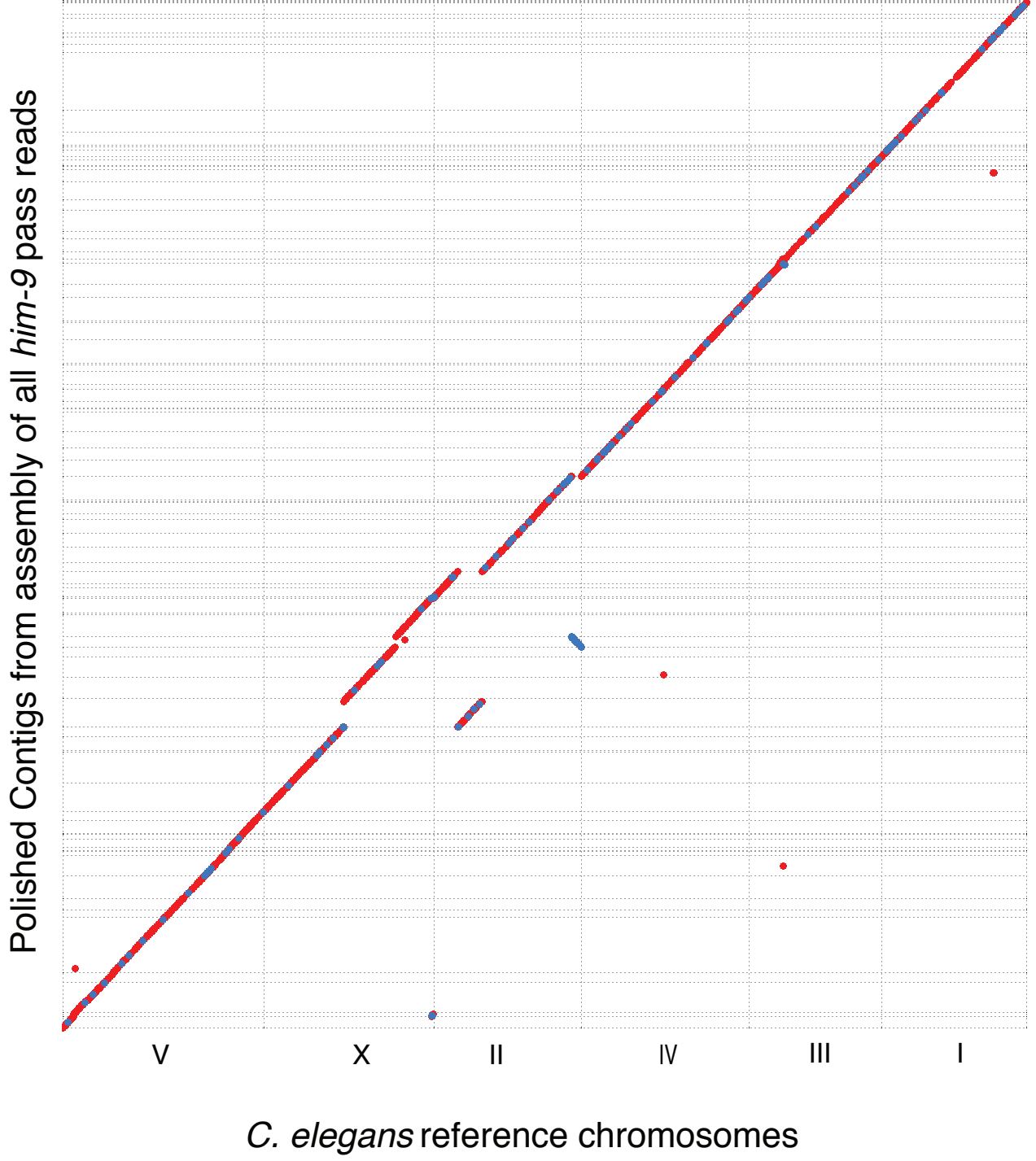

Supplement: Supplemental Material [file supp_gr.221184.117_Supplemental_Fig_S4.pdf]
