# Supplementary material for: MinION-based long-read sequencing and assembly extends the Caenorhabditis elegans reference genome
Source: Genome Res. 2018 Feb;28(2):266–74. doi: 10.1101/gr.221184.117 (PMC5793790; doi:10.1101/gr.221184.117)
Supplement: Supplemental Material [file supp_gr.221184.117_Supplemental_Legends.docx]

**Supplemental Table S1: Library and MinION flowcell run information.** Details of Sample and MinION flowcell runs used to produce data for assembly of the VC2010 Wildtype and *him-9(e1487); ruIs32* genomes.

**Supplemental Table S2: Mummer report extracted stats of different assemblies compared to the *C. elegans* wild type reference sequence GCF_000002985.6_WBcel235.** Reference sequence length = 100,286,401 bases. Albacore 0.8.4 (Non-homopolymer basecalling), Albacore 1.0.1 (Homopolymer basecalling) software (Oxford Nanopore Technologies).

**Supplemental Table S3: Unique mapping contig assembly information from Mummer for the Wild type VC2010 and *him-9(e1487); ruIs32* genomes.** The All data and Chips114+115 data assemblies shown for VC2010.

**Supplemental Table S4: Mummer report extracted stats of different assemblies compared to the *C. elegans* wild type reference sequence GCF_000002985.6_WBcel235.**Reference sequence length = 100,286,401 bases. Albacore 0.8.4 (Non-homopolymer basecalling), Albacore 1.0.1 (Homopolymer basecalling) software (Oxford Nanopore Technologies).

**Supplemental Fig S1: MinION generated read data and wild type *C. elegans* reference coverage.** (A) Plot of % identity for q10 reads aligned to the *C. elegans* reference genome vs read length. (B) All q10 reads mapped to the *C. elegans* reference genome using the BWA aligner resulted in ~ 60 fold coverage.

**Supplemental Fig S2: Bacterial genome assembly identification.** BLAST dot plot of contig014 against the *Stenotrophomonas maltophilia* R551-3 genome.

**Supplemental Fig S3: *him-9(e1487);ruIs32* genome sequencing.** All ‘pass’ reads from *him-9(e1487); ruIs32* sequencing mapped to the *C. elegans* reference genome using the BWA aligner resulted in ~45 fold coverage. Note the large duplication in chromosome IIIR.

**Supplemental Fig S4: *him-9(e1487);ruIs32* genome assembly contiguity.** Mummer alignment plot of contigs from the genome assembly of combined ‘pass’ data from all *him-9* flowcells against the *C. elegans* reference genome demonstrating overall structural agreement. *C. elegans* chromosomes are arranged along the x-axis and *him-9* assembly contigs along the y-axis. Forward strand matches are in red and reverse strand matches are in blue.
