# Supplementary material for: MinION-based long-read sequencing and assembly extends the Caenorhabditis elegans reference genome
Source: Genome Res. 2018 Feb;28(2):266–74. doi: 10.1101/gr.221184.117 (PMC5793790; doi:10.1101/gr.221184.117)
Supplement: Supplemental Material [file supp_gr.221184.117_Supplemental_Table_S3.docx]

|  |  |  | ***C. elegans* Genome** | | **Read Length Stats (kb)** | | |
| --- | --- | --- | --- | --- | --- | --- | --- |
| **Chip #** | **# reads (K)** | **# bases (Mb)** | **Estimated**  **Coverage (fold)** | **Worm Contigs** | **Average** | **Median** | **N50** |
| 112 | 287 | 3,758 | ~38 | 97 | 13 | 13 | 17 |
| 114 | 43 | 894 | ~9 | 421 | 21 | 15 | 38 |
| 115 | 253 | 4,209 | ~42 | 71 | 17 | 17 | 24 |
| 112_114 | 331 | 4,652 | ~47 | 71 | 14 | 13 | 19 |
| 112_115 | 540 | 7,966 | ~80 | 63 | 15 | 14 | 20 |
| 114_115 | 296 | 5,103 | ~51 | 48 | 17 | 17 | 25 |
| All | 583 | 8,8607 | ~89 | 56 | 15 | 14 | 21 |
| All_10K | 377 | 7,915 | ~79 | 65 | 21 | 19 | 22 |
| All_15K | 279 | 6,681 | ~67 | 60 | 24 | 22 | 24 |
| All_20K | 176 | 4,876 | ~49 | 63 | 28 | 26 | 27 |

Supplemental Table 3: Summary of Canu assemblies of *C. elegans* VC2010 wild type strain.
